# Characterization of Acute Myeloid Leukemia With t(16;21) Translocation: Cytogenetic, Molecular, and Immunophenotypic Findings

**DOI:** 10.14740/wjon2700

**Published:** 2026-03-05

**Authors:** Milder Bravo-Davila, Dayana Espinoza-Rodrigez, Javier Orejon-Huarancca, Richard Junior Zapata Dongo

**Affiliations:** a School of Human Medicine, Universidad de Piura, Lima, Peru; b School of Human Medicine, Universidad Nacional Mayor de San Marcos, Lima, Peru

**Keywords:** Leukemia myeloid acute, Core binding factors, RUNX1 translocation partner 1 protein, TLS-ERG fusion protein human, Prognosis

## Abstract

**Background:**

Acute myeloid leukemia (AML) with t(16;21) translocation is an infrequent hematological neoplasia. This study aimed to describe the cytogenetic, molecular and immunophenotypic profiles of this disease.

**Methods:**

We conducted a descriptive observational study using secondary data. AML cases with the t(16;21) translocation were identified from the Mitelman database and systematic searches in PubMed, Scopus, SciELO and Genetics and Cytogenetics in Oncology and Hematology databases. Cytogenetic, molecular, immunophenotypic and clinical variables were extracted. We performed descriptive and survival statistical analyses at 2 and 5 years.

**Results:**

We identified 103 cases with AML with t(16;21). Most cases were t(16;21)(p11;q22) (n = 90, 87.4%), with recurrent additional abnormalities including +10 (14.4%), –16 (7.8%), add(11) (5.6%), and del(6) (4.4%), while t(16;21)(q24;q22) cases mainly showed +8 (45.5%) and del(9) (18.2%). *FUS*::*ERG* was reported in 62.2% of t(16;21)(p11;q22) cases, whereas *RUNX1*::*RUNX1T3* was detected in 72.2% of t(16;21)(q24;q22). Immunophenotypically, t(16;21)(p11;q22) cases expressed (among the cases evaluated) cluster of differentiation (CD)13 (100%), CD33 (96.6%), CD34 (98.0%), CD56 (93.0%), and MPO (84.8%), while all evaluated t(16;21)(q24;q22) cases were positive for CD13, CD33, CD34, and MPO. Relapse information was missing for a substantial proportion of cases; among those with available data (65.0%), relapse occurred in 51 cases (76.1%). The 5-year mortality rate was significantly higher in the t(16;21)(p11;q22) group than in the t(16;21)(q24;q22) group (P = 0.012), with no significant difference at 2 years.

**Conclusions:**

The cytogenetic, molecular, and immunophenotypic characteristics of AML with t(16;21) vary according to the chromosomal breakpoint. The t(16;21)(p11;q22) translocation was the most frequently reported and was frequently associated with CD56 expression. The findings suggest that patients with t(16;21)(p11;q22) exhibited lower 5-year survival compared with the other group, highlighting the unfavorable outcomes observed in reported cases.

## Introduction

Acute myeloid leukemia (AML) is a hematologic neoplasm characterized by the clonal expansion of immature myeloid cells (blasts) that have lost their normal differentiation capacity in the bone marrow [1]. It accounts for approximately 20% of acute leukemia cases in the pediatric population and about 80% in adults [2]. Owing to the biological heterogeneity, the classification of AML has evolved from the French–American–British (FAB) morphological system, which focuses on blast maturation [3], to the current classifications of the World Health Organization (WHO) [4] and the International Consensus Classification (ICC) [5], which prioritize molecular and genetic alterations with respect to morphological findings.

The WHO classifies the types of AML into two main categories: 1) those with defined genetic alterations; and 2) those defined by differentiation. Among the former are those that affect the core binding factor (CBF); these are among the most relevant types of AML because CBF is an essential transcription factor for hematopoietic differentiation. CBF is composed of three core binding factor α (CBFA) subunits that bind DNA (RUNX1, RUNX2 and RUNX3) and a common subunit, core binding factor β (CBFB), which does not bind directly to DNA but potentiates the binding of the other subunits [2]. Physiologically, RUNX1—also known as AML1 or CBFA2—and CBFB form a heterodimer that regulates gene expression during hematopoiesis [6]. However, chromosomal structural alterations can occur in the regions that contain the genes that encode these proteins and therefore can favor the emergence of AML.

The most studied alterations that affect CBF are t(8;21)(q22;q22.1) and t(16;16)(p13.1;q22)/inv(16)(p13.1q22), which give rise to the fusion genes *RUNX1*::*RUNX1T1* and *CBFB*::*MYH11*, respectively [7, 8]. These alterations are associated with favorable prognosis and patient outcomes, with higher complete remission rates after induction therapy and long-term survival rates between 50% and 65% [2, 9].

In addition to these two alterations, the t(16;21) translocation can also compromise the function of CBF, since the *RUNX1* and *CBFB* genes are located on chromosomes 16 and 21. The ICC [5] recognized two additional breakpoints in AML with other rare recurring translocations: t(16;21)(p11;q22), which gives rise to the chimeric protein FUS::ERG [10], which induces leukemia in experimental models with umbilical cord cells [11]; and t(16;21)(q24;q22), which generates the RUNX1::RUNX1T3 fusion protein, which is very similar to the RUNX1::RUNX1T1 protein [12].

Evidence suggests that t(16;21)(q24;q22) AML shares clinical and prognostic characteristics with CBF-AML; in contrast, t(16;21)(p11;q22) AML is associated with poor outcomes, refractoriness to treatment and a high frequency of relapses [13–15]. However, the data available on both fusions are limited and distributed among isolated reports and have great clinical heterogeneity, which has prevented the establishment of a consensus on their biological and prognostic differences. To our knowledge, few systematic efforts have been undertaken to perform a comparative analysis of breakpoint locations based on globally reported cases with AML with t(16;21). These differences appear to constitute a key source of variability in the clinical response among cases with AML.

This study aimed to characterize the cytogenetic, molecular and immunophenotypic profiles of AML with t(16;21); and to explore whether the p11;q22 and q24;q22 breakpoints influence 2- and 5-year survival.

## Materials and Methods

### Design and sources of information

We conducted a descriptive observational study through the analysis of secondary sources of AML cases with t(16;21) reported in scientific articles. For this purpose, we reviewed information from the Mitelman database of Chromosome Aberrations and Gene Fusions [16], sponsored by the National Cancer Institute (United States), the Swedish Cancer Society, and the Swedish Foundation against Childhood Cancer. The database contains data manually extracted from the literature by Mitelman et al [16].

Additionally, to obtain information from as many cases as possible, we searched articles indexed in PubMed, Scopus, and SciELO, as well as those published in the journal of the Atlas of Genetics and Cytogenetics in Oncology and Hematology (AGCOH).

### Participants

Cases with AML with t(16;21) included in the study were those whose data were published in scientific articles without distinction of article type, study design, language, or date. All reported cases with available information on the immunophenotypic profile and cytogenetic and molecular alterations were included, without restrictions on age or sex. Survival data were optional and were not used as exclusion criteria. We excluded cases of therapy-related AML and secondary AML arising from antecedent hematologic disorders, as these entities are classified by the WHO as distinct disease categories with different biological and clinical characteristics [4]. Cases whose full articles could not be retrieved despite exhaustive attempts to contact the corresponding authors were also excluded.

### Variables

The variables correspond to the cytogenetic, molecular and immunophenotypic profiles of the reported cases. At the cytogenetic level, we identified 1) chromosomal breakpoints (16 and 21) with information on the affected band, region and arm; and 2) secondary cytogenetic abnormalities (numerical or structural), which were manually classified on the basis of the karyotype of the case according to the International System for Human Cytogenomic Nomenclature [17].

Molecular alterations were evaluated according to their appearance, primarily FUS::ERG and RUNX1::RUNX1T3, as those are produced by t(16;21), and other reported alterations. Finally, regarding the immunophenotypic profile, we determined whether the cluster of differentiation (CD) evaluated was positive or negative for each case.

Additionally, we recorded other clinical and outcome variables, such as age and age group (pediatric if < 18 years, adult between 18 and 59 and older adult ≥ 60), sex, morphological classification according to FAB (M0: AML with minimal differentiation; M1: AML without maturation; M2: AML with maturation; M3: acute promyelocytic leukemia; M4: acute myelomonocytic leukemia; M5: acute monoblastic or monocytic leukemia; M6: acute erythroid leukemia; M7: acute megakaryoblastic leukemia; and AML not otherwise specified (NOS) [3], type of treatment received (chemotherapy, chemotherapy plus bone marrow transplant, and others), occurrence of AML relapse, overall survival time (in months from diagnosis; in cases where time was recorded in other units, the corresponding conversion was performed: from years to months (multiplying by 12), from days to months (dividing by 30)) and survival at 2 and 5 years from diagnosis (according to outcome: alive or deceased).

### Procedures

We searched for information on the cases with AML with t(16;21) reported as of October 31, 2024. In the Mitelman database, the only filters applied were “abnormality: t(16;21)” and “morphology: acute myeloid leukemia (all subtypes)” in the section “cases cytogenetics”, after which we accessed the article available in PubMed, in which the case was reported through the hyperlink registered for each case in the “case details” section.

Regarding the other data sources, we developed specific search strategies based on keywords and Boolean operators, which are detailed here (Supplementary Material 1, wjon.elmerpub.com). The results of the searches were imported into the Rayyan platform for identifying duplicates. On the same platform, two authors (DE and JO) subsequently performed the screening by reviewing the titles and abstracts independently; if discrepancies were encountered, a third reviewer served as the adjudicator (MB). The full texts of the articles that met the inclusion criteria were evaluated by the authors to determine whether the article should be incorporated into the study. To extract the data of the reported cases, we developed an *ad hoc* electronic form in the REDCap platform.

### Statistical analysis

Data were analyzed using the statistical software STATA V.17.0. Descriptive analyses included reporting medians and interquartile ranges (IQRs) for numerical variables, while categorical variables were expressed as frequencies and percentages. We did not perform inferential analyses because of the descriptive nature of this study.

Kaplan–Meier plots were constructed to describe 2- and 5-year patient survival among individuals for whom follow-up information was available in the publication; the event of interest was patient death.

We presented the secondary structural cytogenetic alterations in a Circos graph prepared with RStudio version 2025.05.1+513 (BioCircos package). Only the chromosomes involved were plotted, without specifying exact locations, as information on affected base pairs within each chromosomal arm region was unavailable for the cases analyzed. All alterations other than the translocation were represented as involving the entire chromosome.

### Ethical aspects

This study was approved by the Institutional Research Ethics Committee of the Universidad de Piura (protocol No. T0624-10) and conducted in compliance with the ethical standards of the responsible institution on human subjects as well as with the Helsinki Declaration. As no individuals were directly evaluated and only data reported in scientific publications were used, the informed consent process was not required.

## Results

**Figure 1:**
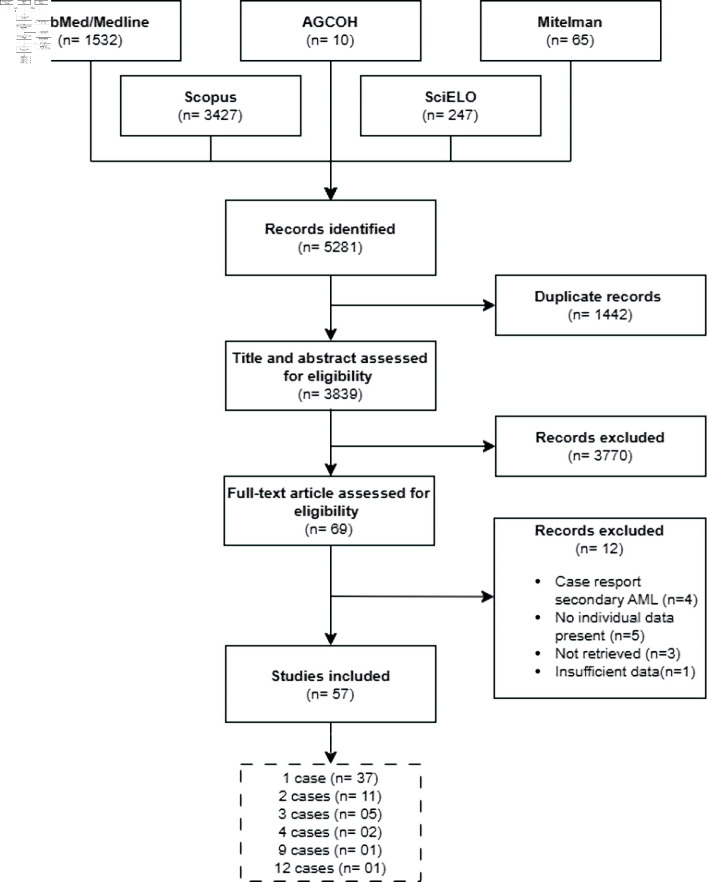
Flowchart of article selection. AML: acute myeloid leukemia; AGCOH: Atlas of Genetics and Cytogenetics in Oncology and Hematology.

**Figure 2:**
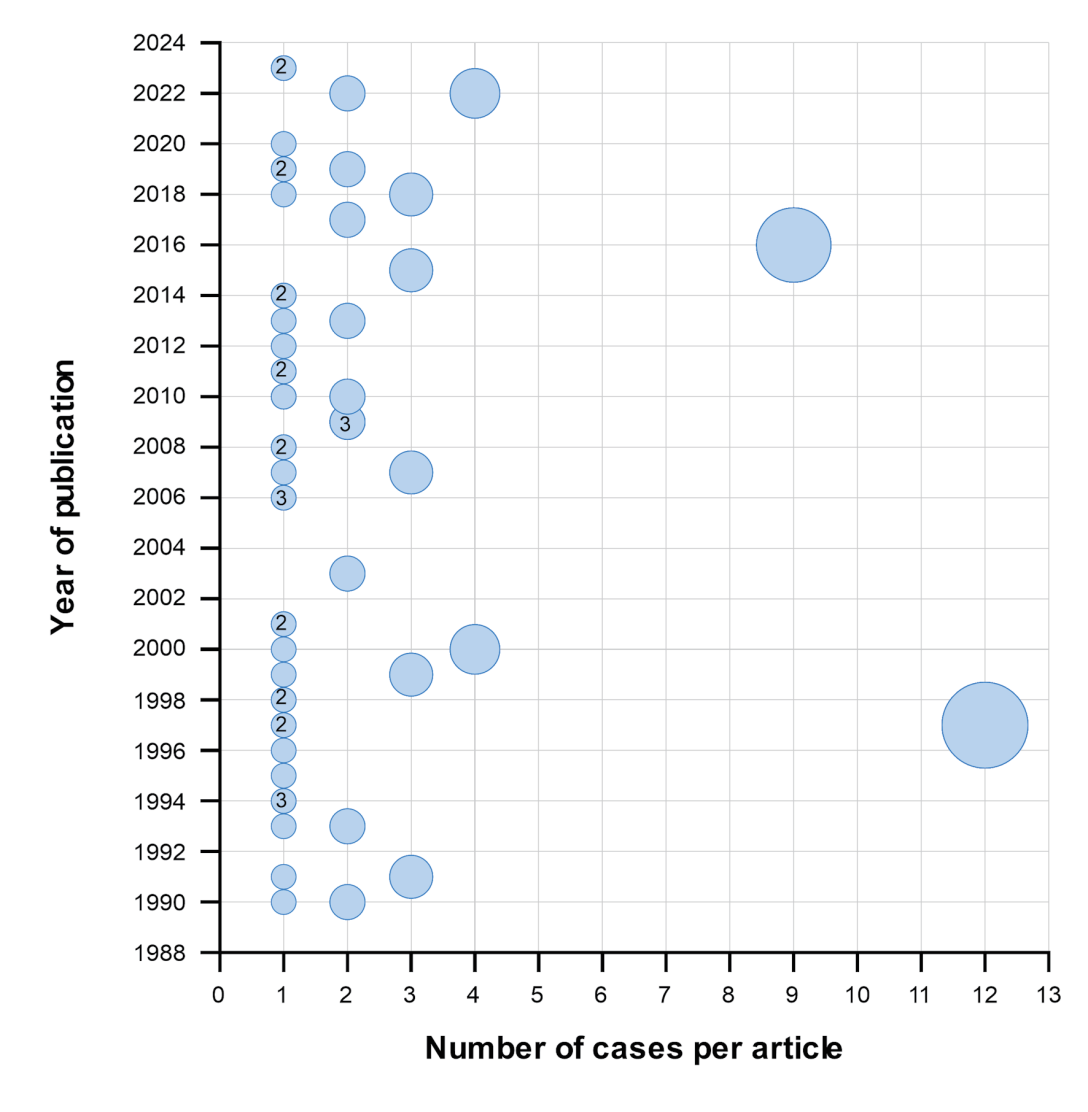
Number of cases reported in each article according to year of publication. Circles without numbers indicate that only one article was published in that year, whereas numbers within circles indicate the number of articles (more than one) published in that year. The size of each circle represents the number of cases reported per article.

We retrieved 5,281 articles from electronic databases. After eliminating duplicates, we evaluated 3,839 articles by their titles and abstracts. Afterward, we excluded 3,770 articles that did not meet the eligibility criteria. We reviewed the remaining 69 full-text articles and included 57 that met the selection criteria (Fig. 1). The selected articles were published between 1990 and 2023 (Fig. 2; Supplementary Material 2, wjon.elmerpub.com). The majority of these (n = 37, 64.9%) reported a single case of AML with t(16;21), for a total of 103 cases.

We observed four chromosomal breakpoints in AML with t(16;21): the most common were t(16;21)(p11;q22) (n = 90, 87.4%) and t(16;21)(q24;q22) (n = 11, 10.7%). Additionally, we identified two infrequent breakpoints, reported in only one case each: t(16;21)(p11;q13) and t(16;21)(q21;p21). The majority of cases were male (n = 56, 54.4%), and the median age was 23 years (IQR: 13–32); the age group distribution differed significantly between groups (P = 0.009), showing age-related differences by chromosomal breakpoint. The most frequent FAB subtypes were M2, M1 and M5, which together represented approximately 70% of the cases. However, no statistically significant association was identified between FAB classification and chromosomal breakpoint. Similarly, no significant differences were observed between both breakpoint groups in the number of chromosomes or in the proportion of cases with numerical or structural chromosomal abnormalities (Table 1).

**Table 1:** General Characteristics of AML Cases With t(16;21) According to Chromosomal Breakpoints

Characteristic	Overall^a^ (n = 103), n (%)	t(16;21) breakpoints		P value
		(p11;q22) (n = 90), n (%)	(q24;q22) (n = 11), n (%)	
Sex (male)	56 (54.4)	50 (55.6)	6 (54.5)	1.000^c^
Age (years)^b^	23 (9–39)	24 (10.5–40.5)	6 (4–71)	0.250^d^
Age group				0.009^c^
Pediatric (< 18 years)	44 (42.7)	37 (41.1)	7 (63.6)	
Adult (18–59 years)	49 (47.6)	46 (51.1)	1 (9.1)	
Older adult (≥ 60 years)	8 (7.8)	5 (5.6)	3 (27.3)	
No information	2 (1.9)	2 (2.2)	0 (0.0)	
FAB classification				0.326^c^
M0: AML with minimal differentiation	1 (1)	1 (1.1)	0 (0.0)	
M1: AML without maturation	22 (21.4)	20 (22.2)	2 (18.2)	
M2: AML with maturation	25 (24.3)	21 (23.3)	4 (36.4)	
M4: acute myelomonocytic leukemia	16 (15.5)	12 (13.3)	4 (36.4)	
M5: acute monoblastic/monocytic leukemia	22 (21.4)	20 (22.2)	0 (0.0)	
M6: acute erythroid leukemia	1 (1)	1 (1.1)	0 (0.0)	
M7: acute megakaryoblastic leukemia	8 (7.8)	8 (8.9)	0 (0.0)	
NOS: AML not otherwise specified	1 (1)	1 (1.1)	0 (0.0)	
No information	7 (6.8)	6 (6.7)	1 (9.1)	
Presence of numerical alterations (yes)	39 (37.9)	32 (35.6)	7 (63.6)	0.101^c^
Number of chromosomes				0.379^c^
< 46	6 (5.8)	6 (6.7)	0 (0.0)	
46	78 (75.7)	69 (76.7)	7 (63.6)	
> 46	17 (16.5)	13 (14.4)	4 (36.4)	
No information	2 (1.9)	2 (2.2)	0 (0.0)	
Presence of structural alterations (yes)	47 (45.6)	40 (44.4)	6 (54.5)	0.542^c^
Primary molecular alterations				< 0.001^c^
RUNX1::RUNX1T3	8 (7.8)	NA	8 (72.7)	
FUS::ERG	56 (54.4)	56 (62.2)	NA	
No information	39 (37.9)	34 (37.8)	3 (27.3)	
Treatment				0.002^c^
Chemotherapy	53 (51.5)	47 (52.2)	6 (54.5)	
Chemotherapy + transplant	30 (29.1)	28 (31.1)	0 (0.0)	
Other	2 (1.9)	0 (0.0)	2 (18.2)	
No information	18 (17.5)	15 (16.7)	3 (27.3)	
Relapse				0.008^c^
Present	51 (49.5)	49 (54.4)	1 (9.1)	
Absent	16 (15.5)	12 (13.3)	3 (27.3)	
No information	36 (35.0)	29 (32.2)	7 (63.6)	

^a^Two additional cases were identified: the first case was in an adult, 46,XX, with t(16;21)(p11:q13) FAB-M5 who received chemotherapy plus transplantation and experienced relapse; the second case was in an adult, 46,XX, with t(16;21)(q21;p21) FAB-M5 with secondary structural chromosomal alterations who received chemotherapy plus transplantation and did not experience relapse. ^b^Median (IQR). ^c^Fisher’s exact test. ^d^Mann–Whitney U test. FAB: French–American–British; M5: acute monoblastic or monocytic leukemia; IQR: interquartile range; NA: not applicable; t(16;21): translocation between chromosomes 16 and 21.

With respect to treatment, chemotherapy alone was the most frequently reported approach in both breakpoint groups; however, treatment distributions differed between breakpoint groups (P = 0.002). Relapses were more frequent (54.4%) in t(16;21)(p11;q22) than in t(16;21)(q24;q22) (9.1%) (P = 0.008), but relapse data were incomplete in 35.0% of the sample (Table 1).

**Figure 3:**
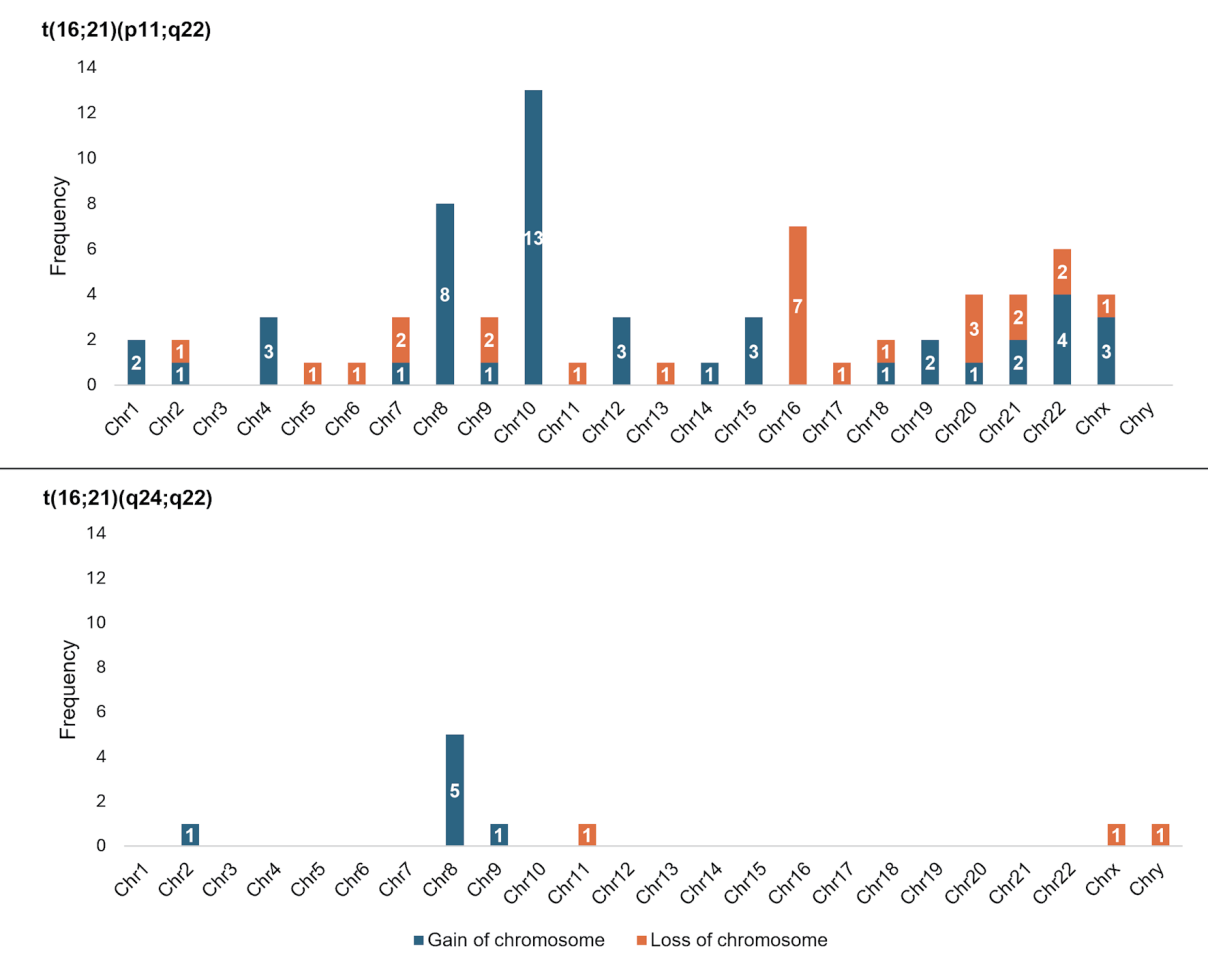
Frequency of numerical chromosomal alterations among cases with AML with t(16;21) according to chromosomal breakpoints. AML: acute myeloid leukemia; t(16;21): translocation between chromosomes 16 and 21; Chr: chromosome.

We identified numerical chromosomal abnormalities in 37.9% of cases (39/103). Among 90 cases with t(16;21)(p11;q22), the most frequent alterations were the gain of chromosome 10 (“+10”; n = 13/90, 14.4%) and the loss of chromosome 16 (“–16”; n = 7/90, 7.8%), both of which were exclusive to this group of cases. While, among 11 cases with t(16;21)(q24;q22), the most frequent alteration was the gain of chromosome 8 (“+8”; n = 5/11, 45.5%), which was proportionately more common than among the t(16;21)(p11;q22) group (n = 8/90, 8.9%). Regarding sex chromosomes, among cases with t(16;21)(p11;q22), 3.3% (n = 3/90) presented with a gain of X (“+X”), and 1.1% (n = 1/90) presented with a loss of X (“–X”). In contrast, among the t(16;21)(q24;q22) group, one case presented with a loss of X (“–X”) (n = 1/11, 9.1%), and another presented with a loss of Y (“–Y”) (n = 1/11, 9.1%) (Fig. 3).

**Figure 4:**
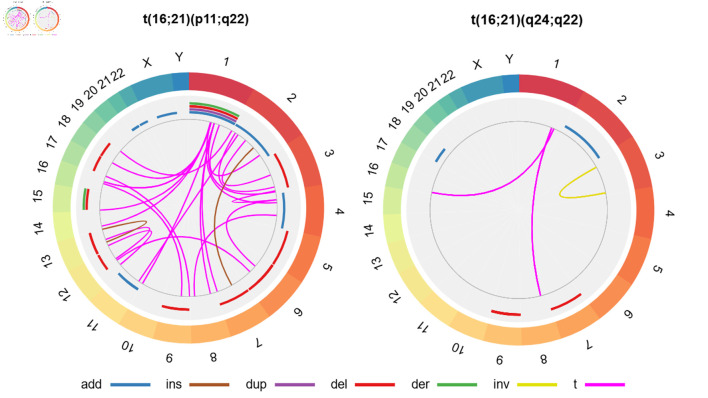
Secondary structural chromosomal alterations in cases with AML with t(16;21) according to chromosomal breakpoints. In the external ring, the chromosomes are individually colored and arranged in clockwise order from chromosome 1 to chromosome Y. In the other rings, chromosomal abnormalities are presented; given that we do not have specific information on the affected chromosomal regions, we present the data as the entire chromosome affected. The sole case with AML with t(16;21)(q21;p21) presented add(17)(q22) and t(1;3)(q24;p24), whereas the case with AML with t(16;21)(p11;q13) showed no secondary abnormalities. The affected arms, regions and bands are shown here (Supplementary Material 3, wjon.elmerpub.com). add: chromosomal addition; ins: insertion; dup: duplication; del: deletion; der: derivative; inv: inversion; t: translocation; AML: acute myeloid leukemia; t(16;21): translocation between chromosomes 16 and 21.

We found that 47 cases (45.6%) had secondary structural chromosomal abnormalities. Among t(16;21)(p11;q22), the most frequent alterations were additions to chromosome 11 (“add(11)”; n = 5/90, 5.6%) and deletions from chromosome 6 (“del(6)”; n = 4/90, 4.4%), whereas among the t(16;21)(q24;q22) group, the most frequent alteration was a deletion from chromosome 9 (“del(9)”; n = 2/11, 18.2%) (Fig. 4; Supplementary Material 3, wjon.elmerpub.com).

Regarding secondary molecular alterations, in the t(16;21)(p11;q22) group, 7.8% of cases (n = 7/90) harbored additional gene mutations, with a total of 12 mutations identified across eight genes, including *DNMT3A*, *BCOR*, *UBA2-WTIP*, *KRAS*, *PHIP-NUP153*, *ASXL1*, *RUNX1*, and *GATA2*. Among these patients, one presented three concurrent mutations, two cases harbored two mutations each, and the remaining four cases exhibited a single mutation. In contrast, in the t(16;21)(q24;q22) group, one case also presented with a mutation in the *RUNX1* gene (9.1%) (Supplementary Material 4, wjon.elmerpub.com).

**Figure 5:**
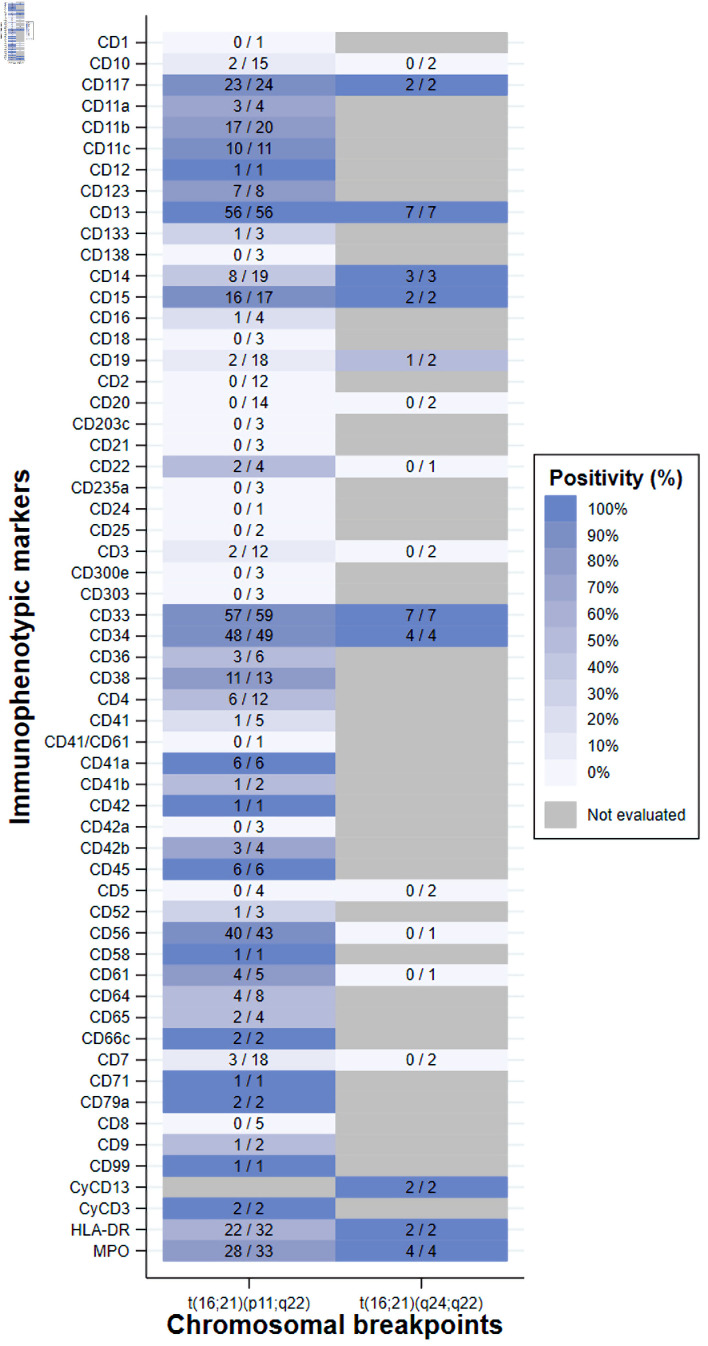
Heatmap of the frequency of immunophenotypic marker positivity among cases with AML with t(16;21). Each cell color in the heatmap represents the proportion of cases with a positive result for a specific immunophenotypic marker, considering only those in whom that marker was assessed (numerator: positive cases; denominator: total cases evaluated). The case with t(16;21)(q21;p21) was positive for CD34, CD38 and HLA-DR, whereas no marker information was available for the case with t(16;21)(p11;q13). AML: acute myeloid leukemia; HLA-DR: human leukocyte antigen-DR; t(16;21): translocation between chromosomes 16 and 21.

Additionally, the expression of immunophenotypic markers was not uniformly evaluated among the studies. Among the 103 cases, the most frequently evaluated markers were CD33 (n = 66, 64.1%), CD13 (n = 63, 61.2%), CD34 (n = 53, 51.5%), CD56 (n = 44, 42.7%) and myeloperoxidase (MPO) (n = 37, 35.9%). Percentages were calculated as the proportion of cases in which each marker was evaluated relative to the total number of cases. In the t(16;21)(p11;q22) group, the most frequent positive immunophenotypic markers (among the cases evaluated) were CD33 (57/59, 96.6%), CD13 (56/56, 100%), CD34 (48/49, 98.0%), CD56 (40/43, 93.0%) and MPO (28/33, 84.8%). Percentages were calculated as the proportion of marker-positive cases relative to the total number of cases evaluated for each marker. In the t(16;21)(q24;q22) group, the expression of the markers CD13 (7/7), CD33 (7/7), CD34 (4/4) and MPO (4/4) was positive in all cases evaluated (Fig. 5).

**Figure 6:**
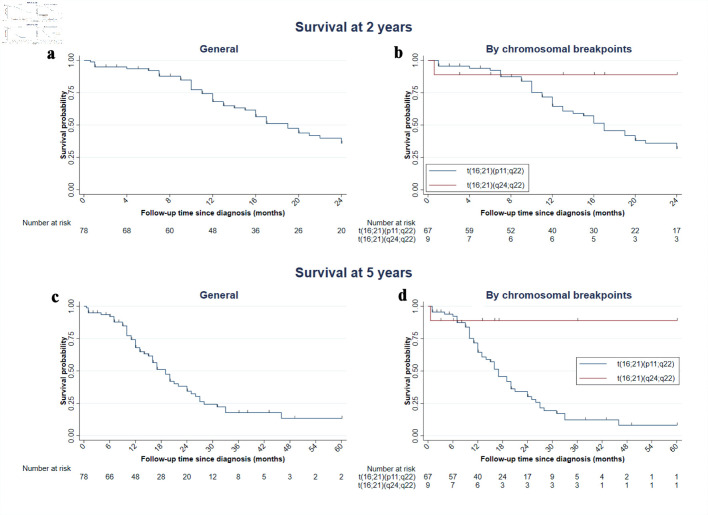
Kaplan–Meier graph of survival at 2 and 5 years in cases with AML with t(16;21). Of the 103 cases, 78 had survival information available. (a, c) Overall survival at 2 and 5 years; this includes the case with t(16;21)(q21;p21), with a follow-up time of 14 months, and the case with t(16;21)(p11;q13), who died at 22 months. (b, d) Curves stratified by translocation subtype. AML: acute myeloid leukemia; t(16;21): translocation between chromosomes 16 and 21.

Finally, we conducted an exploratory survival analysis including 78 cases (75.7%) with available survival time data. Of these, 40 patients (52.3%) died within the first 2 years of follow-up, with a median survival of 19 months (IQR: 15–24), yielding a mortality rate of 35.9 per 1,000 person-years (95% confidence interval (CI): 26.3–48.9). However, no statistically significant difference in survival at 2 years was observed between the t(16;21)(p11;q22) and t(16;21)(q24;q22) breakpoint groups (P = 0.062). Among the 50 cases with available 5-year mortality data, all patients died, with a median survival of 19 months (95% CI: 11–28), corresponding to a mortality rate of 37.4 per 1,000 person-years. In the comparison of survival across chromosomal breakpoints, a statistically significant difference was observed between t(16;21)(p11;q22) and t(16;21)(q24;q22) at 5 years (P = 0.012) (Fig. 6; Supplementary Material 5, wjon.elmerpub.com).

## Discussion

Among the 103 cases with AML with t(16;21) reported in the literature, the chromosomal breakpoint location t(16;21)(p11;q22) was predominant, observed in 87.4% of the cases. This is consistent with a pediatric cohort study that reported a similar proportion [13]. The t(16;21)(q24;q22) cases we obtained were few, possibly because these cases were seen more frequently in secondary AML [18]. Therefore, the comparisons presented in this study should be interpreted within the context of *de novo* AML and may not be fully extrapolated to secondary or therapy-related AML cases harboring t(16;21).

The other two chromosomal breakpoints identified may represent incidental findings, as the original case reports did not describe the fusion genes generated by these translocations. Nevertheless, their inclusion in future studies is warranted to clarify their potential leukemogenic role and to better characterize the features.

In addition to the aforementioned translocations, secondary chromosomal alterations—both numerical and structural abnormalities in addition to t(16;21)—were also reported and will be discussed below.

One of the secondary numerical chromosomal alterations was trisomy 10, which was observed exclusively in cases with t(16;21)(p11;q22), similar to findings reported in a pediatric cohort [13]. In contrast, trisomy 8 was present in cases with either breakpoint, with a particularly high frequency in cases with t(16;21)(q24;q22) (45.5%), similar to that observed in a pediatric cohort (36.8%) [13]. These findings suggest that certain chromosomal gains could play a cooperative role in the pathogenesis of AML with t(16;21).

Similar anomalies have also been documented in AML-CBF. In our opinion, it is important to consider possible points of convergence and divergence between AML-CBF and AML with t(16;21), especially the t(16;21)(q24;q22) breakpoint. In this context, trisomy 8 was also frequently observed (12%) in a previous study on AML-CBF [19], which could reflect some biological convergence between the two groups. However, in our study, we did not identify other secondary numerical anomalies similar to those reported in AML-CBF [19, 20]. In contrast, to our knowledge, trisomy 10, which was found exclusively in cases with t(16;21)(p11;q22), has not been identified as a recurrent alteration in AML-CBF [19, 20]. These findings suggest that t(16;21)(p11;q22) could represent a biological entity with distinct characteristics.

Another secondary numerical alteration reported in AML-CBF is the loss of sex chromosomes [19, 20]. Surprisingly, in our study, we identified only three of these chromosomal events, which represented 2.9% of the cases. Compared with the characteristics of AML-CBF in cases from the United States and Europe, the loss of sex chromosomes is common in cases with t(8;21) (loss of Y in 44% of males and of X in 37% of females). However, in cases with inv(16)/t(16;16), loss of the Y chromosome has been observed in only 5% of males [19]. Although we detected a low frequency of these alterations, this similarity suggests that AML-CBF and t(16;21) share chromosomal rearrangement mechanisms that could involve different biological processes. Therefore, future research should evaluate the role of these alterations in survival and other clinical outcomes in cases with this disease.

A total of 45.6% of the cases had secondary structural chromosomal abnormalities. This value is comparable to that described in other series, where the prevalence of complex karyotypes was between 26% and 38% [13, 18]. To our knowledge, the role of these alterations in survival and other outcomes remains uncertain; therefore, it is essential to conduct analytical studies to determine their impact. For example, in AML-CBF with t(8;21), the presence of del(9q) has been associated with refractory disease and worse disease progression [21]. In our study, we also found del(9) in some cases with t(16;21)(q24;q22), which highlights the need for additional studies to define its clinical relevance.

Most primary studies did not systematically apply the tests necessary for detecting secondary molecular alterations. Among the few alterations identified, mutations involved in methylation processes, chromatin remodeling, transcription factors and the RTK-RAS signaling pathway have been identified [22]; the latter is the most frequently affected in cases with t(16;21)(p11;q22), as reported by one study [23]. In the literature, AML-CBF with mutations in *RTK-RAS* genes, such as *C-KIT* [24] and *FLT3-ITD*, are associated with poor outcomes [22, 24, 25], whereas *ASXL1* mutations are associated with favorable outcomes [22]. However, owing to the nature of our study and the limited number of cases, we could not analyze the implications of these mutations in cases with AML with t(16;21) or evaluate whether there is any similarity with what has been reported for AML-CBF.

The immunophenotypes of the cases consistently confirmed the myeloid origin of their diseases, with high expression of CD13, CD33 and CD117. In addition, some demonstrated relatively high expression of monocytic markers (CD11c, CD14, CD36 and CD64), which is consistent with that described in AML [4]. The frequent expression of CD34 reflects blastemic immaturity, although it lacks lineage specificity [26], which explains why it is the third most frequent immunophenotype among cases with either chromosomal breakpoint. Additionally, we found a high frequency of positive expression of CD56, MPO and CD15, which are the most frequent markers of AML-CBF [27]. This similarity, consistent with that proposed in a previous study [28], nevertheless requires further study. The high frequency of CD56 positivity in the t(16;21)(p11;q22) group coincides with the findings of a previous study, in which all cases expressed this marker [29]. This is important since CD56 is associated with poor outcomes in cases with AML in general and specifically in those with AML with t(8;21) [30]. These findings could help explain the unfavorable outcomes described for cases with t(16;21)(p11;q22).

Our review revealed a high proportion of cases without available information on relapse. Among cases with t(16;21)(p11;q22), more than half experienced relapse at least once, which is consistent with the findings of previous studies reporting frequencies of 67.7% and 78.6% [13, 18]. This high recurrence may be attributable to the presence of the FUS::ERG fusion protein, which has been shown to promote *in vitro* pharmacological resistance to azacitidine after repeated exposures [31]. On the other hand, in the t(16;21)(q24;q22) group, we found a low relapse proportion; however, 63.6% of the cases did not report information. In two other studies, the relapse proportion was found to range between 0% and 33.3% [13, 18]. In AML-CBF, meanwhile, the proportion of recurrence has been reported to be between 31.1% [19] and 69.4% [20]. However, the high proportion of cases with missing relevant information precludes the formulation of robust hypotheses regarding differences in relapse between t(16;21)(q24;q22) and t(16;21)(p11;q22).

Finally, survival differed markedly according to the breakpoint. We found that cases with t(16;21)(p11;q22) were more likely to experience early mortality, which is consistent with the results of a study in which the median overall survival was 18.2 months [23]. In contrast, cases with t(16;21)(q24;q22) had more favorable survival, similar to that reported in a pediatric cohort [13]. Descriptively, these findings highlight the relevance of the chromosomal breakpoint in predicting patient outcomes; however, inferential studies are needed to clarify the contribution of this variable and others to survival outcomes.

This study has several limitations. First, we did not search for cases in gray literature sources; however, we did include large-coverage databases. Second, the analysis relied on published reports, and additional cases identified in clinical practice may remain unreported. Moreover, primary studies did not uniformly report key variables such as immunophenotypic or molecular analyses, treatment regimens, or relapse status, resulting in the lack of information for a considerable proportion of cases. The ELN22 risk stratification could not be applied because it requires mandatory molecular mutations and complete cytogenetic alterations, which were not consistently available. In addition, follow-up duration varied across studies, limiting the interpretation of survival analysis. Treatment was not included as a covariate, and variability in therapeutic approaches—together with the inclusion of older reports that may not reflect current therapeutic advances—may have influenced outcomes, as treatment data were not reported in a standardized manner. Additionally, in the Circos plot, we depicted the entire chromosome as affected because we did not have exact information on where the chromosomal alterations occurred. Despite these limitations, this study provides a comprehensive characterization of AML with t(16;21) and may serve as a foundation for future research.

In conclusion, AML with t(16;21) is a rare and heterogeneous entity characterized by distinct cytogenetic, molecular, and immunophenotypic features according to the chromosomal breakpoint. In our study, the t(16;21)(p11;q22) translocation was the most frequent. Additionally, secondary chromosomal abnormalities were frequently observed, with trisomy 8 and trisomy 10 being the most common. Notably, trisomy 10 was identified exclusively in cases with t(16;21)(p11;q22), further supporting the presence of underlying biological differences between AML cases harboring the two breakpoints.

With regard to molecular alterations, no definitive conclusions could be drawn, as these abnormalities were reported in only a small proportion of cases and were not comparable across studies. Concerning immunophenotypic characteristics, as expected, myeloid lineage markers were the most frequently expressed. However, cases with t(16;21)(p11;q22) showed a higher expression of the unfavorable immunophenotypic marker CD56, whereas t(16;21)(q24;q22) displayed immunophenotypic features more similar to those observed in AML with core-binding factor rearrangements.

Although survival and relapse analyses were not the primary focus of this study, the observed differences in clinical outcomes suggest that the chromosomal breakpoint may have prognostic relevance. Overall, these findings emphasize the importance of detailed cytogenetic characterization and highlight the need for multicenter studies with standardized case reporting to further elucidate the biological and clinical implications of AML with t(16;21).

## Supplementary Material

Suppl 1 Search strategies.

Suppl 2 Articles included in the review.

Suppl 3 Frequencies of secondary structural chromosomal abnormalities.

Suppl 4 Distribution of additional gene mutations in patients with t(16;21).

Suppl 5 Overall survival at 2 and 5 years by t(16;21) chromosomal breakpoint.

## Data Availability

The data supporting the findings of this study are available from the corresponding author upon reasonable request.

## Abbreviations

t(16;21): translocation between chromosomes 16 and 21
AML: acute myeloid leukemia
CD: cluster of differentiation
FAB: French–American–British
WHO: World Health Organization
ICC: International Consensus Classification
CBF: core binding factor
CBFA: core binding factor α
CBFB: core binding factor β
IQR: interquartile range
AGCOH: Atlas of Genetics and Cytogenetics in Oncology and Hematology
NOS: not otherwise specified
NA: not applicable
Chr: chromosome
HLA-DR: human leukocyte antigen-DR
MPO: myeloperoxidase
RUNX1: Runt-related transcription factor 1
RUNX2: Runt-related transcription factor 2
RUNX3: Runt-related transcription factor 3
*RUNX1*::*RUNX1T3*: fusion gene formed by *RUNX1* (Runt-related transcription factor 1) and *RUNX1T3* (RUNX1 translocation partner 3)
*RUNX1*::*RUNX1T1*: fusion gene formed by *RUNX1* (Runt-related transcription factor 1) and *RUNX1T1* (RUNX1 translocation partner 1)
*CBFB*::*MYH11*: fusion gene formed by *CBFB* (core binding factor subunit β) and *MYH11* (myosin heavy chain 11)
*FUS*::*ERG*: fusion gene formed by *FUS* (fused in sarcoma) and *ERG* (ETS-related gene)
M0: AML with minimal differentiation
M1: AML without maturation
M2: AML with maturation
M3: acute promyelocytic leukemia
M4: acute myelomonocytic leukemia
M5: acute monoblastic or monocytic leukemia
M6: acute erythroid leukemia
M7: acute megakaryoblastic leukemia
AML-NOS: acute myeloid leukemia, not otherwise specified
ASXL1: additional sex combs-like 1
BCOR: BCL6 corepressor
DNMT3A: DNA (cytosine-5)-methyltransferase 3A
ETS: E26 transformation-specific (family of transcription factors)
UBA2: ubiquitin-like modifier activating enzyme 2
WTIP: Wilms tumor 1 interacting protein
KRAS: Kirsten rat sarcoma viral oncogene homolog
GATA2: GATA binding protein 2
NUP153: nucleoporin 153
PHIP: Pleckstrin homology domain-interacting protein
RTK–RAS: receptor tyrosine kinase–RAS signaling pathway
KIT (c-KIT): receptor tyrosine kinase encoded by the *KIT* gene (V-kit Hardy–Zuckerman 4 feline sarcoma viral oncogene homolog)
FLT3-ITD: FMS-like tyrosine kinase 3 internal tandem duplication
