# Supplementary material for: Characterization of Acute Myeloid Leukemia With t(16;21) Translocation: Cytogenetic, Molecular, and Immunophenotypic Findings
Source: World J Oncol. 2026 Mar 5;17(2):178–90. doi: 10.14740/wjon2700 (PMC12978396; doi:10.14740/wjon2700)
Supplement: Suppl 1 — Search strategies. [file wjon-17-02-178-s001.docx]

**Supporting information**

**Suppl 1. Search Strategies**

| **Database** | **Search strategy** | **Results (Search performed on October 31, 2024)** |
| --- | --- | --- |
| AGCOH | t(16;21) | 10 articles |
| PubMed | (("Leukemia, Myeloid, Acute" [Mesh]) OR (Acute myeloid leukemia [Title/Abstract]) OR (LMA [Title/Abstract])) AND ((t(16;21) [Title/Abstract]) OR (t(16;21)(q24;q22) [Title/Abstract]) OR (t(16;21)(p11;q22) [Title/Abstract]) OR (FUS-ERG [Title/Abstract]) OR (CBFA2T3 [Title/Abstract]) OR (ETO2 [Title/Abstract]) OR (MTG16 [Title/Abstract]) OR (MTGR2 [Title/Abstract]) OR (RUNX1T3 [Title/Abstract]) OR (ZMYND4 [Title/Abstract]) OR (CBFA2 [Title/Abstract]) OR (RUNX1 [Title/Abstract]) OR (RUNX1-RUNX1T3 [Title/Abstract])) | 1532 articles |
| Scopus | TITLE-ABS-KEY ( "acute myeloid leukemia" OR "leukemia, myeloid, acute" OR "AML" ) AND TITLE-ABS-KEY ( "t(16;21)" OR "t(16;21)(q24;q22)" OR "t(16;21)(p11;q22)" OR "FUS-ERG" OR "CBFA2T3" OR "ETO2" OR "MTG16" OR "MTGR2" OR "RUNX1T3" OR "ZMYND4" OR "CBFA2" OR "RUNX1" OR "RUNX1-RUNX1T3" ) | 3427 articles |
| SciELO | (ab:((ti:(LMA)) OR (ab:(LMA)) OR (ti:(leucemia mieloide aguda)) OR (ab:(leucemia mieloide aguda)) AND (ti:(t(16;21))) OR (ab:(t(16;21))) OR (ti:(t(16;21)(q24;q22))) OR (ab:(t(16;21)(q24;q22))) OR (ti:(t(16;21)(p11;q22))) OR (ab:(t(16;21)(p11;q22))) OR (ti:(FUS-ERG)) OR (ab:(FUS-ERG)) OR (ti:(RUNX1T3)) OR (ab:(RUNX1T3)))) | 247 articles |

AGCOH: Atlas of Genetics and Cytogenetics in Oncology and Hematology
