# Supplementary material for: Characterization of Acute Myeloid Leukemia With t(16;21) Translocation: Cytogenetic, Molecular, and Immunophenotypic Findings
Source: World J Oncol. 2026 Mar 5;17(2):178–90. doi: 10.14740/wjon2700 (PMC12978396; doi:10.14740/wjon2700)
Supplement: Suppl 2 — Articles included in the review. [file wjon-17-02-178-s002.docx]

**Supporting information**

**Suppl 2. Articles Included in the Review**

| **N°** | **Identifier (DOI)*** | **Author** | **Year of publication** | **Title of the article** | **Number of cases included** |
| --- | --- | --- | --- | --- | --- |
| 1 | 10.1159/000205069 | Sadamori, N. et al. | 1990 | 1621 translocation in acute nonlymphocytic leukemia with abnormal eosinophils: a unique subtype | 2 |
| 2 | 10.1016/0165-4608(90)90274-e | Berkowicz, M. et al. | 1990 | Acute nonlymphocytic leukemia with t(1621) | 1 |
| 3 | 10.1016/0165-4608(91)90117-d | Morgan, R. et al. | 1991 | t(1621)(p11.2q22): a recurrent primary rearrangement in ANLL | 3 |
| 4 | 10.1016/0165-4608(91)90030-x | Marosi, C. et al. | 1991 | Translocation (1621)(p11q22) in acute monoblastic leukemia with erythrophagocytosis | 1 |
| 5 | 10.1016/0165-4608(93)90187-q | Nobbs, M.C. et al. | 1993 | Acute non-lymphocytic leukemia with t(1621) | 1 |
| 6 | 10.1182/blood.v81.6.1573.1573 | Maseki, N. et al. | 1993 | The 821 chromosome translocation in acute myeloid leukemia is always detectable by molecular analysis using AML1 | 2 |
| 7 | 10.1182/blood.v84.1.244.244 | Scott, A. et al. | 1994 | HLA-DR-, CD33+, CD56+, CD16- myeloid/natural killer cell acute leukemia: a previously unrecognized form of acute leukemia potentially misdiagnosed as French-American-British acute myeloid leukemia-M3 | 1 |
| 8 | PMID: 8139114 | Satoh, K. et al. | 1994 | [Acute monoblastic leukemia (M5a) with dysmegakaryocytopoiesis associated with t(1621) (p11q22)] | 1 |
| 9 | 10.1016/0165-4608(94)90216-x | Okada, K. et al. | 1994 | Translocation (1621)(p11q22) in acute nonlymphocytic leukemia | 1 |
| 10 | 10.1111/j.1365-2141.1995.tb05168.x | Hiyoshi, M. et al. | 1995 | Establishment and characterization of IRTA17 and IRTA21, two novel acute non-lymphocytic leukaemia cell lines with t(1621) translocation | 1 |
| 11 | 10.1002/(SICI)1096-8652(199612)53:4<264::AID-AJH12>3.0.CO2-C | Ohshima, A. et al. | 1996 | 11q23 aberration is an additional chromosomal change in de novo acute leukemia after treatment with etoposide and mitoxantrone | 1 |
| 12 | 10.1038/sj.leu.2400594 | Yamamoto, K. et al. | 1997 | Establishment of a novel human acute myeloblastic leukemia cell line (YNH-1) with t(1621), t(116) and 12q13 translocations. | 1 |
| 13 | https://doi.org/10.1182/blood.V90.3.1192 | Kong, X. et al. | 1997 | Consistent detection of TLS/FUS-ERG chimeric transcripts in acute myeloid leukemia with t(1621)(p11q22) and identification of a novel transcript. | 12 |
| 14 | 10.1620/tjem.183.297 | Harigae, H. et al. | 1997 | Detection of Minimal Residual Disease in Cerebro-Spinal Fluid of a Patient with Acute Myelogenous Leukemia with t(16 21)(p11 q22) Translocation by Reverse Transcriptase-Polymerase Chain Reaction | 1 |
| 15 | 10.1046/j.1365-2141.1998.00735.x | Straetmans, N. et al. | 1998 | Haemopoietic defect and decreased expansion potential of bone marrow autografts from patients with acute myeloid leukaemia in first remission | 1 |
| 16 | 10.3892/ijo.12.6.1259 | Tamura, S. et al. | 1998 | Cytogenetic analysis of de novo acute myeloid leukemia with trilineage myelodysplasia in comparison with myelodysplastic syndrome evolving to acute myeloid leukemia. | 1 |
| 17 | 10.1046/j.1365-2141.1999.01384.x | Shikami, M. et al. | 1999 | Myeloid differentiation antigen and cytokine receptor expression on acute myelocytic leukaemia cells with t(1621)(p11q22): frequent expression of CD56 and interleukin-2 receptor alpha chain. | 3 |
| 18 | https://www.scopus.com/pages/publications/0033161272 | Imashuku, S. et al. | 1999 | Hemophagocytosis by leukemic blasts in a case of acute megakaryoblastic leukemia with t(1621)(p11q22) | 1 |
| 19 | 10.1002/(SICI)1097-0142(20000415)88:8<1970::AID-CNCR28>3.0.CO2-9 | Imashuku, S. et al. | 2000 | Hemophagocytosis by leukemic blasts in 7 acute myeloid leukemia cases with t(1621)(p11q22): Common morphologic characteristics for this type of leukemia | 4 |
| 20 | 10.1016/S0145-2126(99)00137-X | Okita, H. et al. | 2000 | Acute myeloid leukemia possessing jumping translocation is related to highly elevated levels of EAT/mcl-1, a Bcl-2 related gene with anti-apoptotic functions | 1 |
| 21 | 10.1159/000046532 | Okoshi, Y. et al | 2001 | Detection of minimal residual disease in a patient having acute myelogenous leukemia with t(1621)(p11q22) treated by allogeneic bone marrow transplantation. | 1 |
| 22 | PMID: 11505530 | Fukushima, Y. et al | 2001 | [AML(M7) associated with t(1621)(p11q22) showing relapse after unrelated bone marrow transplantation and disappearance of TLS/FUS-ERG mRNA]. | 1 |
| 23 | PMID: 12673593 | Wu, Y.-F. et al | 2003 | Clinical and experimental studies on five cases of acute myeloid leukemia with translocation t(1621) (p11q22) | 2 |
| 24 | 10.1016/j.cancergencyto.2006.01.003 | Choi, H.-W. et al | 2006 | Unusual type of TLS/FUS-ERG chimeric transcript in a pediatric acute myelocytic leukemia with 47,XX,+10,t(1621)(p11q22). | 1 |
| 25 | 10.1016/j.cancergencyto.2005.08.005 | Jeandidier, E. et al. | 2006 | Abnormalities of the long arm of chromosome 21 in 107 patients with hematopoietic disorders: a collaborative retrospective study of the Groupe Français de Cytogénétique Hématologique | 1 |
| 26 | https://atlasgeneticsoncology.org/case-report/208829/a-case-of-trisomy-8-and-loss-of-the-y-chromosome-as-secondary-aberrations-in-a-ten-year-old-boy-with-de-novo-aml-fab-m2-and-t(16;21)(q24;q22) | Bradtke, J. et al. | 2007 | A case of trisomy 8 and loss of the Y-chromosome as secondary aberrations in a ten year old boy with de novo AML FAB M2 and t(1621)(q24q22) | 1 |
| 27 | 10.1111/j.1600-0609.2007.00854.x | Betts, D.R. et al. | 2007 | The prognostic significance of cytogenetic aberrations in childhood acute myeloid leukaemia. A study of the Swiss Paediatric Oncology Group (SPOG) | 2 |
| 28 | 10.1016/j.leukres.2006.10.010 | Zatkova, A. et al. | 2006 | A patient with de novo AML M1 and t(1621) with karyotype evolution. | 1 |
| 29 | 10.1016/j.cancergencyto.2008.04.011 | De Braekeleer, É. et al. | 2008 | RUNX1-MTG16 fusion gene in acute myeloblastic leukemia with t(1621)(q24q22): case report and review of the literature. | 1 |
| 30 | 10.1159/000117710 | Boils, C. et al. | 2008 | T(1621)(q24q22) in acute myeloid leukemia: Case report and review of the literature | 1 |
| 31 | 10.1016/j.cancergencyto.2009.06.010 | Kim, J. et al. | 2009 | Detection of FUS-ERG chimeric transcript in two cases of acute myeloid leukemia with t(1621)(p11.2q22) with unusual characteristics. | 2 |
| 32 | 10.3343/kjlm.2009.29.5.390 | Chang, W. et al. | 2009 | [Two cases of acute myeloid leukemia with t(1621)(p11q22) and TLS/FUS-ERG fusion transcripts]. | 2 |
| 33 | 10.1073/pnas.0903142106 | Radtke, I. et al. | 2009 | Genomic analysis reveals few genetic alterations in pediatric acute myeloid leukemia. | 2 |
| 34 | 10.1016/j.cancergencyto.2009.08.017 | Park, I. et al. | 2010 | Acute myeloid leukemia with t(1621)(q24q22) and eosinophilia: case report and review of the literature. | 1 |
| 35 | 10.1016/j.cancergencyto.2010.01.005 | Schmidt-Hieber, M. et al. | 2010 | Cytogenetic studies in acute leukemia patients relapsing after allogeneic stem cell transplantation. | 1 |
| 36 | 10.1016/j.cancergencyto.2010.04.009 | Oh, S. et al. | 2010 | Two childhood cases of acute leukemia with t(16;21)(p11.2;q22): second case report of infantile acute lymphoblastic leukemia with unusual type of FUS-ERG chimeric transcript | 1 |
| 37 | 10.3109/10428194.2010.522286 | Athanasiadou, A. et al. | 2011 | RUNX1-MTG16 fusion gene in de novo acute myeloblastic leukemia with t(1621)(q24q22). | 2 |
| 38 | 10.1016/j.cancergen.2011.02.005 | Jiang, H. et al. | 2011 | Establishment and characterization of a novel acute myeloid leukemia cell line, JIH-4, carrying a t(1621)(p11.2q22) and expressing the FUS-ERG fusion. | 1 |
| 39 | 10.1111/j.1743-7563.2011.01427.x | Manabe, M. et al. | 2011 | Transient efficacy of cord blood transplantation in acute myeloid leukemia with t(1621)(p11q22) | 1 |
| 40 | 10.1007/s12185-012-1044-7 | Kawashima, N. et al. | 2012 | Childhood acute myeloid leukemia with bone marrow eosinophilia caused by t(1621)(q24q22). | 1 |
| 41 | 10.1038/ng.2759 | Yoshida, K. et al. | 2013 | The landscape of somatic mutations in Down syndrome-related myeloid disorders | 1 |
| 42 | 10.1007/s12185-013-1495-5 | Ismael, O. et al. | 2014 | RUNX1 mutation associated with clonal evolution in relapsed pediatric acute myeloid leukemia with t(1621)(p11q22). | 2 |
| 43 | 10.2169/internalmedicine.53.1275 | Ozeki, K. et al. | 2014 | Relapse of acute myeloid leukemia mimicking autoimmune pancreatitis after bone marrow transplantation | 1 |
| 44 | 10.1038/leu.2014.72 | Niu, X. et al. | 2014 | Acute myeloid leukemia cells harboring MLL fusion genes or with the acute promyelocytic leukemia phenotype are sensitive to the Bcl-2-selective inhibitor ABT-199 | 1 |
| 45 | 10.3892/ol.2015.3051 | Zhang, Z. et al. | 2015 | Clinical characteristics and laboratory analyses of acute myeloid leukemia with t(1621)(p11q22) | 3 |
| 46 | 10.3760/cma.j.issn.0253-2727.2016.03.007 | Ouyang, M. et al. | 2016 | [Clinical characteristics of acute myeloid leukemia with t (1621) (p11q22):nine cases report and literature review]. | 9 |
| 47 | 10.1111/ijlh.12714 | Kobayashi, K. et al. | 2017 | CD66c (KOR-SA3544) antigen expression of leukemic blasts in pediatric acute myeloid leukemia with TLS/FUS-ERG fusion transcript. | 3 |
| 48 | 10.1007/s00277-018-3267-z | Zerkalenkova, E. et al. | 2018 | Molecular characteristic of acute leukemias with t(1621)/FUS-ERG. | 3 |
| 49 | 10.1016/j.yexcr.2018.08.035 | Lu, X. et al. | 2018 | Identification of the UBA2-WTIP fusion gene in acute myeloid leukemia | 1 |
| 50 | 10.3389/fonc.2019.01358 | Yao, S. et al. | 2019 | Donor-Derived CD123-Targeted CAR T Cell Serves as a RIC Regimen for Haploidentical Transplantation in a Patient With FUS-ERG+ AML. | 1 |
| 51 | 10.1002/ccr3.2461 | Keino, D. et al. | 2019 | Salvage therapy with azacitidine for pediatric acute myeloid leukemia with t(1621)(p11q22)/FUS-ERG and early relapse after allogeneic blood stem cell transplantation: A case report. | 1 |
| 52 | 10.24875/BMHIM.20000025 | Saucedo-Campos, A. et al. | 2020 | Acute myeloid leukemia associated with t(16:21)(p11q22) in a pediatric patient. | 1 |
| 53 | 10.1007/s00277-022-04979-5 | Zhang, H. et al. | 2022 | TLS/FUS-ERG fusion gene in acute leukemia and myelodysplastic syndrome evolved to acute leukemia: report of six cases and a literature review. | 4 |
| 54 | 10.1038/s41467-022-29336-y | Liu, T. et al. | 2022 | Distinct genomic landscape of Chinese pediatric acute myeloid leukemia impacts clinical risk classification | 2 |
| 55 | 10.7759/cureus.42215 | Teshima, K. et al. | 2023 | Acute Myeloid Leukemia Harboring the t(1621)(p11q22) Translocation Treated With Venetoclax Plus Azacitidine After Cord Blood Transplantation | 1 |
| 56 | 10.1007/s12185-023-03580-4 | Fukushima, H. et al. | 2023 | Acute pancreatitis as the initial manifestation of acute myeloid leukemia with chromosome 16 rearrangements | 1 |
| 57 | https://doi.org/10.1182/bloodadvances.2019000404 | Shiba, N. et al. | 2019 | Transcriptome analysis offers a comprehensive illustration of the genetic background of pediatric acute myeloid leukemia | 2 |

***Most articles have a DOI identifier; however, some only have a PubMed ID (PMID) or article link.**
