# Supplementary material for: Characterization of Acute Myeloid Leukemia With t(16;21) Translocation: Cytogenetic, Molecular, and Immunophenotypic Findings
Source: World J Oncol. 2026 Mar 5;17(2):178–90. doi: 10.14740/wjon2700 (PMC12978396; doi:10.14740/wjon2700)
Supplement: Suppl 3 — Frequencies of secondary structural chromosomal abnormalities. [file wjon-17-02-178-s003.docx]

**Supporting information**

**Suppl 3. Frequencies of Secondary Structural Chromosomal Abnormalities**

| **Structural chromosomal alteration** | **Alteration of the chromosome, arm, region and specific band** | **Breakpoints of t(16;21)** | | **Overall * N=103 n (%)** |
| --- | --- | --- | --- | --- |
|  |  | **t(16;21)(p11;q22) N=90**  **n (%)** | **t(16;21)(q24;q22) N=11**  **n (%)** |  |
| +del(1) | +del(1)(q21):+del(1) (q42) | 1 (1.1) | 0 (0) | 1 (1) |
| +del(6) | +del(6)(q13) | 1 (1.1) | 0 (0) | 1 (1) |
| add(1) | add(1)(p13) | 1 (1.1) | 0 (0) | 1 (1) |
|  | add(1)(q?) | 1 (1.1) | 0 (0) | 1 (1) |
| add(11) | add(11)(q23) | 1 (1.1) | 0 (0) | 1 (1) |
|  | add(11)(p?) | 1 (1.1) | 0 (0) | 1 (1) |
|  | add(11)(q13) | 2 (2.2) | 0 (0) | 2 (1.9) |
|  | add(11)(q?) | 1 (1.1) | 0 (0) | 1 (1) |
| add(17) | add(17)(q22) | 0 (0) | 0 (0) | 1 (1) |
| add(18) | add(18)(p11.2) | 0 (0) | 1 (9.1) | 1 (1) |
| add(2) | add(2)(q31) | 0 (0) | 1 (9.1) | 1 (1) |
|  | add(2)(q?) | 1 (1.1) | 0 (0) | 1 (1) |
| add(21) | add (21)(q22) | 2 (2.2) | 0 (0) | 2 (1.9) |
|  | add(21)(q?) | 1 (1.1) | 0 (0) | 1 (1) |
| add(22) | add(22)(p11) | 1 (1.1) | 0 (0) | 1 (1) |
| add(4) | add(4)(q?) | 1 (1.1) | 0 (0) | 1 (1) |
| add(X) | add(X)(q22) | 1 (1.1) | 0 (0) | 1 (1) |
| del(1) | del(1)(p13) | 1 (1.1) | 0 (0) | 1 (1) |
| del(12) | del(12)(p13) | 1 (1.1) | 0 (0) | 1 (1) |
| del(13) | del(13)(q12q14) | 2 (2.2) | 0 (0) | 2 (1.9) |
| del(15) | del(15)(q11.2q15) | 1 (1.1) | 0 (0) | 1 (1) |
| del(17) | del(17)(?) | 1 (1.1) | 0 (0) | 1 (1) |
| del(18) | del(18)(p11.2) | 1 (1.1) | 0 (0) | 1 (1) |
| del(3) | del(3)(p21) | 1 (1.1) | 0 (0) | 1 (1) |
| del(5) | del(5)(q22;q34) | 1 (1.1) | 0 (0) | 1 (1) |
| del(6) | del(6)(q?) | 1 (1.1) | 0 (0) | 1 (1) |
|  | del(6)(q21) | 2 (2.2) | 0 (0) | 2 (1.9) |
|  | del(6)(q13) | 1 (1.1) | 0 (0) | 1 (1) |
| del(7) | del(7)(q?) | 1(1.1) | 1 (9.1) | 2 (1.9) |
| del(9) | del(9)(q22q33) | 0 (0) | 1 (9.1) | 1 (1) |
|  | del(9)(?) | 2 (2.2) | 0 (0) | 2 (1.9) |
|  | del(9)(q22q34) | 0 (0) | 1 (9.1) | 1 (1) |
|  | del(9)(q12q22) | 1 (1.1) | 0 (0) | 1 (1) |
| der(1) | der(1)(?) | 1 (1.1) | 0 (0) | 1 (1) |
| +der(1;7) | +der(1;7)(q10;p10) | 1 (1.1) | 0 (0) | 1 (1) |
| der(15) | der(15)(?) | 1 (1.1) | 0 (0) | 1 (1) |
| dup(1) | dup(1)(q21q42) | 1 (1.1) | 0 (0) | 1 (1) |
| ins(13;13) | ins(13,13)(q14;q22q34) | 1 (1.1) | 0 (0) | 1 (1) |
| ins(7;2) | ins(7;2)(q11;p14p23) | 1 (1.1) | 0 (0) | 1 (1) |
| inv(3) | inv(3)(p23q26) | 0 (0) | 1 (9.1) | 1 (1) |
| t(1;10) | t(1;10)(q21;q26) | 1 (1.1) | 0 (0) | 1 (1) |
| t(1;14) | t(1;14)(p11;p11) | 1 (1.1) | 0 (0) | 1 (1) |
|  | t(1;14)(q10;q32) | 1 (1.1) | 0 (0) | 1 (1) |
| t(1;16) | t(1;16)(q12;q13) | 2 (2.2) | 0 (0) | 2 (1.9) |
|  | t(1;16)(q32;p13.3) | 0 (0) | 1 (9.1) | 1 (1) |
| t(1;17) | t(1;17)(q12;q25) | 1 (1.1) | 0 (0) | 1 (1) |
| t(1;2) | t(1;2)(q25;q33) | 1 (1.1) | 0 (0) | 1 (1) |
| t(1;3) | t(1;3)(q24;p24) | 0 (0) | 0 (0) | 1 (1) |
|  | t(1;3)(q21;q27) | 1 (1.1) | 0 (0) | 1 (1) |
| t(1;4) | t(1;4)(q10;q10) | 1 (1.1) | 0 (0) | 1 (1) |
| t(1;7) | t(1;7)(q21;q35) | 1 (1.1) | 0 (0) | 1 (1) |
| t(1;8) | t(1;8)(q21;p21) | 1 (1.1) | 0 (0) | 1 (1) |
|  | t(1;8)(q31;p21) | 0 (0) | 1 (9.1) | 1 (1) |
| t(12;13) | t(12;13)(q15;q14) | 1 (1.1) | 0 (0) | 1 (1) |
|  | t(12;13)(p11;p11) | 1 (1.1) | 0 (0) | 1 (1) |
| t(2;11) | t(2;11)(p21;p15) | 1 (1.1) | 0 (0) | 1 (1) |
| t(2;18) | t(2;18)(?) | 1 (1.1) | 0 (0) | 1 (1) |
| t(2;3) | t(2;3)(q?;p?) | 1 (1.1) | 0 (0) | 1 (1) |
|  | t(2;3)(p23;q25) | 1 (1.1) | 0 (0) | 1 (1) |
| t(3;4) | t(3;4)(q27;p15) | 1 (1.1) | 0 (0) | 1 (1) |
| t(4;6) | t(4;6)(p14;q11) | 1 (1.1) | 0 (0) | 1 (1) |
| t(4;8) | t(4;8)(q28;q24.1) | 1 (1.1) | 0 (0) | 1 (1) |
| t(6;12) | t(6;12)(q21;q13) | 1 (1.1) | 0 (0) | 1 (1) |
| t(9;16) | t(9;16)(q12;q11) | 1 (1.1) | 0 (0) | 1 (1) |
|  | t(9;16)(?) | 1 (1.1) | 0 (0) | 1 (1) |

*** Includes a patient with t(16;21)(q21;p21) who presented add(17)(q22) and t(1;3)(q24;p24), whereas the patient with t(16;21)(p11;q13) showed no secondary structural alterations. ?: data missing.**
