# Supplementary material for: Characterization of Acute Myeloid Leukemia With t(16;21) Translocation: Cytogenetic, Molecular, and Immunophenotypic Findings
Source: World J Oncol. 2026 Mar 5;17(2):178–90. doi: 10.14740/wjon2700 (PMC12978396; doi:10.14740/wjon2700)
Supplement: Suppl 4 — Distribution of additional gene mutations in patients with t(16;21). [file wjon-17-02-178-s004.docx]

**Suppl 4.** Distribution of additional gene mutations in patients with t(16;21).

| **Case** | **t(16;21) breakpoint** | **Additional gene mutations** | **N° of mutations** |
| --- | --- | --- | --- |
| 1 | t(16;21)(q24;q22) | RUNX1 | 1 |
| 2 | t(16;21)(p11;q22) | DNMT3A, ASXL1 | 2 |
| 3 | t(16;21)(p11;q22) | BCOR, RUNX1, ASXL1 | 3 |
| 4 | t(16;21)(p11;q22) | UBA2-WTIP | 1 |
| 5 | t(16;21)(p11;q22) | BCOR | 1 |
| 6 | t(16;21)(p11;q22) | KRAS | 1 |
| 7 | t(16;21)(p11;q22) | KRAS, GATA2 | 2 |
| 8 | t(16;21)(p11;q22) | PHIP-NUP153 | 1 |
| Each row represents an individual patient. A total of twelve additional mutations across eight genes were identified. RUNX1 listed in this table refers to additional gene mutations and not to the gene involved in the defining chromosomal translocation | | | |
