# Supplementary material for: Characterization of Acute Myeloid Leukemia With t(16;21) Translocation: Cytogenetic, Molecular, and Immunophenotypic Findings
Source: World J Oncol. 2026 Mar 5;17(2):178–90. doi: 10.14740/wjon2700 (PMC12978396; doi:10.14740/wjon2700)
Supplement: Suppl 5 — Overall survival at 2 and 5 years by t(16;21) chromosomal breakpoint. [file wjon-17-02-178-s005.docx]

**Suppl 5.** Overall survival at 2 and 5 years by t(16;21) chromosomal breakpoint

| **Chromosomal breakpoint** | **n** | **Median (IQR)** | **Deaths** | **Mortality rate per 1,000 person-years (95% CI)** | **P Value†** |
| --- | --- | --- | --- | --- | --- |
| Overall survival at 2 years | 78 | 19 (15 - 24) | 40 | 35.86 (26.3; 48.88) |  |
| t(16;21)(p11;q22) | 67 | 17 (13 - 21) | 38 | 39.92 (29.04; 54.86) | 0.062 |
| t(16;21)(q24;q22) | 9 | * | 1 | 7.84 (1.10; 55.64) |  |
| t(16;21)(p11;q13) | 1 | * | 1 | 45.45 (6.4; 322.69) |  |
| t(16;21)(q21;p21) | 1 | * | 0 | * |  |
| Overall survival at 5 years | 50 | 19 (11 - 28) | 50 | 37.41 (28.35; 49.36) |  |
| t(16;21)(p11;q22) | 48 | 17 (11 - 27) | 48 | 43.13 (32.5; 57.23) | 0.012 |
| t(16;21)(q24;q22) | 1 | * | 1 | 5.33 (0.75; 37.84) |  |
| t(16;21)(p11;q13) | 1 | * | 1 | 45.45 (6.4; 322.69) |  |
| t(16;21)(q21;p21) | 0 | * | 0 | * |  |
| † Stratified log-rank test for comparison of survival functions between t(16;21)(p11;q22) and t(16;21)(q24;q22).* Not calculable due to the small number of cases | | | | | |
